# Assessing the pharmaceutical care provision to suspected COVID-19 patients in community pharmacies: a simulated patient study

**DOI:** 10.1186/s12913-022-07870-1

**Published:** 2022-04-09

**Authors:** Samar Karout, Hani M. J. Khojah, Rania Itani, Fatima Jaffal, Abdalla El-Lakany

**Affiliations:** 1 Pharmacy Practice Department, Faculty of Pharmacy, Beirut Arab University, P.O.Box: 11-5020, Riad El Solh, Beirut, 1107 2809 Lebanon; 2 Clinical and Hospital Pharmacy Department, College of Pharmacy, Taibah University, P.O.Box: 30051, Madinah, 41477 Kingdom of Saudi Arabia; 3 Department of Pharmaceutical Sciences, Faculty of Pharmacy, Beirut Arab University, P.O.Box: 11-5020, Riad El Solh, Beirut, 1107 2809 Lebanon

**Keywords:** Simulated patient, Pharmaceutical care, Patient care, COVID-19, Lebanon

## Abstract

**Background:**

In the wake of COVID-19, community pharmacists (CP) were called upon to free up healthcare providers to treat more serious conditions and alleviate overcrowded healthcare centers. CPs were placed under tremendous pressure, where many patients primarily sought their health advice. This situation raised concerns about the preparedness of CPs in facing these challenges. Therefore, this study aimed to assess the appropriateness of pharmaceutical care provided by CPs to patients with suspected COVID-19 and to investigate their communication skills.

**Methods:**

A simulated patient (SP) study was conducted among randomly selected community pharmacies in Beirut, Lebanon. Each pharmacy was visited by the SP who complained of fever and loss of smell sensation. Interactions between the attending pharmacist and the suspected COVID-19 patient were documented directly after each visit in a standardized data collection form.

**Results:**

More than half of the CPs (56%) did not retrieve any relevant information to assess the patient’s condition. While pharmacists’ responses were limited to one to two recommendations, with the majority recommending the patient to perform the PCR test (90%). Inappropriate recommendations made by the CPs included mainly the confirmation that the patient had COVID-19 without prior testing (9%), and prescribing either an antimicrobial drug (5%) or dietary supplements (20%), claiming that the latter are essential to boost the patient’s immunity. As for the pharmacist-patient communication skills, the mean total score was 2.25 ± 0.79 (out of 4), displaying nonoptimal and ineffective communication.

**Conclusion:**

An unsatisfactory and suboptimal provision of pharmaceutical care to a suspected COVID-19 case was evident. This may be a public health threat, particularly for developing countries that lack an efficient and unified healthcare system. The findings should alert health authorities to support and guide community pharmacists in assisting suspected COVID-19 patients.

**Supplementary Information:**

The online version contains supplementary material available at 10.1186/s12913-022-07870-1.

## Introduction

In the wake of the COVID-19 pandemic, healthcare systems around the world have been placed under extreme pressure [1]. The level of preparedness of these systems largely determines the social vulnerability to the spreading of the infection and the capacity for infection control [2]. Developed countries such as the United States, Canada, China, United Kingdom, and Germany have effectively and promptly adapted and responded to their new portfolios by leveraging a wide range of quality pharmaceutical care services [3, 4]. According to the definition developed by Hepler and Strand, pharmaceutical care is “the responsible provision of drug therapy for the purpose of achieving definite outcomes that improve a patient’s quality of life” and it “involves the process through which a pharmacist co-operates with a patient and other professionals in designing, implementing and monitoring a therapeutic plan that will produce specific therapeutic outcomes for the patient” [5]. In the context of the COVID-19 pandemic, the pharmaceutical care services include raising public awareness by providing guidance on preventive measures to minimize the risk of transmission, triaging patients with COVID-19, ensuring a safe supply of COVID-19 vaccines, educating patients about the infection through various media including telehealth services, clarifying misconceptions about COVID-19 treatment development, updating drug formularies, and addressing drug shortages [6].

However, developing countries, particularly those with economic challenges and vulnerable healthcare systems, may have failed to align with the sudden rise of the health care demands [6]. Accordingly, these countries may not be able to adopt the recommended international COVID-19 response strategies to flatten the infection-rate curve [7]. In Lebanon, for example, the pandemic occurred concurrently with a time of particular social and economic turmoil that drained the resources of the healthcare system [6, 8]. Although Lebanon has traditionally been considered to have a relatively robust healthcare system, medical and pharmaceutical services are currently under tremendous pressure.

Community pharmacists (CPs) are among the scarce healthcare personnel who remain reachable in person for patients, although CPs may struggle with limited personal protective equipment (PPE) [9, 10]. CPs remained in the frontline with their pharmacies opened round the clock even during lockdowns [11]. Thus, they were the most accessible and affordable healthcare providers in the pandemic [12, 13]. CPs are uniquely placed to provide services for patients with suspected COVID-19. As such, patients primarily seek medical advice from CPs, who provide consultations free of charge [14, 15]. This situation raised concerns about the preparedness of CPs in facing these challenges solely.

By December 2020, in the wake of the dramatic increase of COVID-19 cases, the Lebanese government declared a public mobilization to close the borders, with a complete lockdown of non-essential services. Flights were canceled and banned to countries with the exponential increase of COVID-19 cases, public transportation was shut down, as well as schools and universities closed their doors [16, 17]. Moreover, the Lebanese healthcare system became saturated as hospitals were unable to attend all COVID-19 patients who required medical care. In the midst of this situation, community pharmacists were available in providing services for patients with suspected COVID-19, especially because there were limited resources available for free screening offered by the government [15, 18]. Many patients primarily sought medical advice from community pharmacists, who provide consultations free of charge [15]. Therefore, in response to this high flow of patients’ visits to community pharmacies, this study was conducted during the second wave of COVID-19, between January and February 2021.

At the time of writing this manuscript, most studies that evaluated the quality of pharmaceutical care services in response to COVID-19 were self-administered questionnaires that are prone to bias and do not reflect the actual behavior of pharmacy staff [15, 19, 20]. However, a recent study from Saudi Arabia, conducted at the beginning of the outbreak using the simulated client technique, has reflected a low level of community pharmacy preparedness for COVID-19 regarding the availability and quality of PPE, health promotional services, and counseling against the outbreak [21]. The use of a simulated patient (SP) methodology has substantially increased in the field of pharmacy practice for the past decade [22]. This method is utilized to evaluate the performance of community pharmacists, assess the quality of pharmaceutical services, and evaluate the impact of interventions [22, 23]. The SP studies reveal the actual pharmacists’ behavior because they are not aware they are being evaluated [22, 24]. Accordingly, the current study aimed to assess the appropriateness of pharmaceutical care provided by Lebanese CPs to patients with suspected COVID-19 and to investigate their communication skills using the SP technique.

## Materials and methods

### Study design

The study was conducted in Beirut, the national capital of Lebanon, during January and February 2021, using the SP technique. A research assistant was trained to play the role of a simulated patient, following a well-structured scenario to identify the actual and spontaneous responses of the community pharmacists. The SP is a female pharmacist who works in a community pharmacy as well as a research assistant and has already participated in several simulated patient studies. She was instructed not to proceed with the role-play if her identity was revealed, and to ask for vitamin C effervescent tablets instead. Moreover, if the pharmacist offered to sell a medication to the SP, the counseling behavior was observed by the SP, who would later pretend that she forgot to bring her wallet and act like she was going home to bring it so that she can pay for the medications. She would then thank the pharmacist and leave.

Finally, the SP captured the time of each visit, using a personal stopwatch, and documented each pharmacist’s responses using the data collection form, immediately after leaving the pharmacy to minimize any possible recall bias.

### Selection of pharmacies

At the time of the study, there were 235 registered community pharmacies in Beirut [25]. Pharmacies were coded and their list was scrambled, and the first 100 pharmacies were selected as a sample using an online sample size calculator from Raosoft®, assuming that appropriate care for COVID-19 suspects is provided by 50% of the pharmacies, with an accepted margin of error of 7.5% and a confidence interval of 95% [26]. A pharmacy would be replaced by the next one from the remaining pharmacies if the SP’s identity was revealed, or the pharmacy was unreachable or found closed.

### The simulated patient scenario

The applied scenario included a 25-year-old female (the SP) who visited the selected pharmacies, adhering to the PPE (wearing a surgical face mask, a face shield, and gloves). She avoided touching surfaces and maintained a safe distance from others in the pharmacy. The SP approached the dispensary, requesting to speak with the pharmacist. The SP, while looking worried about her health, informed the pharmacist that she was experiencing fever and loss of smelling sensation within the past 2 days, and she requested the pharmacist’s health advice with regards to her symptoms, “I’m having fever and loss of smell for the past two days. What should I do?”

The following information was provided only upon the pharmacist’s request. She was unemployed, previously healthy, non-smoker, living with her parents, and was not receiving any chronic medication. She measured her body temperature several times, and it ranged between 38 and 38.5 °C. When asked about the medications utilized to cope with her symptoms, she reported that she took one tablet of Profinal XP® (ibuprofen 400 mg/caffeine 65 mg) 2 h before her visit to manage her fever. She did not have any red flag symptoms such as dyspnea, confusion, chest pain, decreased urine output, hemoptysis, bluish lips, or cold extremities. She visited a friend a week ago, who recently tested positive for COVID-19, and she did not undergo the polymerase chain reaction (PCR) test.

### Data collection form development and structure

A standard form was developed after an extensive literature review by the research team [3, 27–30]. It consisted of five main sections, with 40 different items, varying between close-ended (pre-specified options) and open-ended questions. The first section gathered information about the pharmacy being visited, including the pharmacy code, sex and the estimated age of the encountered pharmacist, the date, time, and duration of the visit.

The second section recorded the questions being asked by the pharmacist to retrieve the patient’s medical history. The third section identified the pharmacist’s response to the patient’s situation. The second and third sections of the data collection form were developed based on the guidance that was issued by the Centers for Disease Control and Prevention (CDC), which guides the healthcare providers to advise suspected COVID-19 patients on seeking appropriate medical care [28]. This guidance includes screening for COVID-19 exposure and symptoms, evaluating life-threatening conditions, assessing high-risk patients, a decision algorithm for appropriate case disposition, and tailored care advice messages.

Finally, the last section assessed the encountered pharmacist’s communication skills using a validated, patient-centered model [29]. This model consisted of several items including nonverbal and verbal communication skills such as eye contact, body posture, active listening, questioning, preparedness and approach, empathy, and closure. Each item was rated on a 4-point scale, ranging from 0 (unsatisfactory performance) to 4 (distinguished performance). Cronbach’s Alpha test was used to assess the internal reliability of the scale, which yielded 0.89, making the scale reliable with good internal consistency.

Additionally, another two experts in pharmacy practice have reviewed the form for face and content validity. An amendment was made based on their recommendations (see Additional File. 1 for the data collection form).

### The SP training

The SP was trained by the study principal investigator (PI) to perform the role-play and use the data collection form. Then, several role-plays, for the above-designed scenario, were performed between SP and another study researcher, acting as a community pharmacist. Meanwhile, the PI was observing and assessing the performance of the SP during the role-plays and giving his feedback. Then both the SP and the PI documented the mock pharmacist’s responses, after each role-play, using the data collection form, and compared their findings to ensure consistency of the recorded observations.

### The pilot test

The SP visited 10 pharmacies (other than the sample) accompanied by one researcher (acting as a client) to validate the simulation process, assess the scenario’s feasibility, and ensure the reliability and reproducibility of the data collection form. Modifications to the scenario and the form were made accordingly.

### Ethical considerations

The World Medical Association Declaration of Helsinki guidance was followed in designing and conducting this study [31]. The protocol was approved by the Institutional Review Board of Beirut Arab University (No. 2020-H-0072-P-M-0438) with a waiver of informed consent for the nature of the study. This waiver did not adversely affect the rights and welfare of the subjects, and all measures to protect their confidentiality were taken. Moreover, the recruited simulated patient (SP) is one of the study authors, so no further ethical parameters were required.

### Data analysis

Data were analyzed by the 24th version of the Statistical Package for the Social Science (SPSS®, IBM Corp., Armonk, NY, USA). The descriptive data were represented by frequencies and percentages for categorical variables, and mean with standard deviation for continuous variables.

## Results

One hundred pharmacies were included in the study. The SP’s identity was revealed in one pharmacy that was replaced by the next one from the reserve list. The mean duration of the visits was 1.98 ± 0.91 min (ranging from 1 to 5 min). Table 1 summarizes the observations found by the SP.

**Table 1:** The simulated patient’s observations during the pharmacy visits

Observation	n (or %)^**a**^
Pharmacist’s age	
*< 40 years*	69
*> 40 years*	31
Pharmacist’s sex	
*Male*	47
*Female*	53
Method of communication	
*Through plexiglass inside the pharmacy*	68
*Face to face inside the pharmacy*	25
*Through dispensing window outside the pharmacy*	7

^a^Since the number of pharmacists is 100, the result indicates the percentage too

### Relevant medical data obtained

Only 44% of the pharmacists obtained relevant medical data from the SP, where 19% of them retrieved only one element. The information obtained by the pharmacists are shown in Table 2.

**Table 2:** Relevant information requested by the pharmacists

Information	n (or %)^**a**^
None	56
Symptoms	34
Onset and duration of symptoms	16
Previous exposure to a confirmed COVID-19 case	15
Previous PCR tests undergone	6
Medications taken to cope with symptoms	5
Experiencing COVID-19 red flag symptoms	3
Patient’s residence	2

*PCR* polymerase chain reaction

^a^Since the number of pharmacists is 100, the result indicates the percentage too. As mixed responses were given, numbers do not add up to 100

### Pharmacist responses to the suspected COVID-19 case

Pharmacist responses were classified into either appropriate or inappropriate, as shown in Table 3. Of the 18% of the pharmacists who advised the SP to take an antipyretic as needed, 16% recommended taking paracetamol and 2% recommended a non-steroidal anti-inflammatory drug. The recommended non-pharmacological means for combating SP’s symptoms were drinking plenty of fluids (3%) and eating soups (2%). In addition, gargling with salt and water, applying cold patches, and eating warm food were recommended by one pharmacist each.

**Table 3:** Pharmacists’ Responses to the Suspected COVID-19 Case

Appropriate responses	n (or %)^**a**^
Recommended undergoing the PCR test	90
Informed the patient that she might have COVID-19	41
Educated the patient about COVID-19	37
Recommended avoidance of contacting others	27
Recommended an antipyretic as needed for fever	18
Advised the patient to monitor her symptoms	9
Advised the patient to isolate herself for 14 days at home	7
Recommended non-pharmacological measure(s)	5
Advised the patient to seek medical attention	4
***Inappropriate responses***	
Recommended a supplement that may not be necessary	20
Confirmed that the patient had COVID-19	9
Recommended other medication (e.g., antibiotics and antihistamines)	5
Assured the patient that she is not infected with COVID-19	2
Informed the patient that undergoing a PCR test is not mandatory	2

*PCR* polymerase chain reaction

^a^Since the number of pharmacists is 100, the result indicates the percentage too. As mixed responses were given, numbers do not add up to 100

Inappropriate recommendations were provided by a variety of pharmacists. Five percent of the pharmacists prescribed a medication that is not indicated for the patient’s condition. These included antibiotics (4%), antihistamines (2%), and pseudoephedrine (1%). In addition, 20% of the pharmacists prescribed some dietary supplements that may not be necessary. These supplements were vitamin C (19%), zinc (18%), vitamin D (12%), and multivitamins (1%). Noticeably, one pharmacist prescribed vitamin D supplement as one tablet daily for 3 months, and then one tablet once a week indefinitely, stating that it is necessary to treat and prevent COVID-19.

### Pharmacists’ communication skills

The mean score (out of 4) achieved by the pharmacists for each communication element is presented in Table 4. The mean total score was 2.25 ± 0.79. The participants’ communication performance outcomes were subdivided into four categories: unsatisfactory (1–1.75; 33%), emerging (1.76–2.51; 31%), proficient (2.52–3.26; 22%), and distinguished (3.27–4; 14%).

**Table 4:** Communication skills scores for the encountered pharmacists

Element	Mean (out of 4) ± SD
Eye contact	2.98 ± 0.83
Non-verbal communication	3.10 ± 1.07
Listening	2.10 ± 1.17
Questions	1.67 ± 1.05
Concern	2.14 ± 1.11
Organization	1.98 ± 1.00
Empathy	1.92 ± 1.12
Closure	2.15 ± 1.02

*SD* standard deviation

## Discussion

While several studies had previously investigated the quality of pharmaceutical care in response to the COVID-19 pandemic, most of them used self-administered questionnaires that are usually susceptible to social desirability bias [32, 33]. At the time of writing, this study was the first to use the simulated patient approach to effectively address the pharmaceutical care provided to suspected COVID-19 cases by Lebanese CPs. The findings showed that participants had an overall unsatisfactory level of preparedness in response to a suspected COVID-19 case. Alarmingly, 56% of these pharmacists did not retrieve any relevant information to assess the patient’s condition, although the Centers for Disease and Prevention Center (CDC) and the International Pharmaceutical Federation (FIP) have issued guidelines for primary healthcare providers to assist them in responding to the COVID-19 outbreak [4, 34, 35]. The current study has revealed a suboptimal provision of pharmaceutical care services, which is in line with several SP studies that explored community pharmacists’ performance and yielded an unsatisfactory assessment, recommendation, and counseling offered to patients [30, 36–38].

In this study, the pharmacists’ response was limited to one to two recommendations, with the majority of them recommending the patient to do the PCR test, which is considered the gold standard recommendation for this patient’s condition [39]. However, only 7% instructed the patient to isolate herself for 14 days. Other inappropriate recommendations made by the CPs included confirmation that the patient had COVID-19 without prior testing, informing the patient that the PCR test is not mandatory, assuring the patient is not infected with COVID-19 and prescribing either an antimicrobial drug or dietary supplements. Despite the controversy about the benefits of dietary supplements in treating or preventing COVID-19 infection [40–43], some encountered pharmacists claimed that dietary supplements are essential to boost the patient’s immunity. Thus, exposing the patient to falsified hyperbolic health claims, lacking rigorous evidence to support their efficacy.

Inappropriate counseling can be viewed as an ethical dispute, where some pharmacists demonstrate their preference for financial gain while violating the patient’s beneficence. This malpractice possesses significant consequences on the patient’s health, profession, reputation, and also the healthcare system [44, 45]. Studies have shown that pharmacists, who provided quality pharmaceutical services, had clarified the misunderstandings about COVID-19, improved patient’s health outcomes, reduced the risk for severe disease, prevented virus transmission, and subsequently decreased healthcare expenditures [34, 46]. Furthermore, proper counseling not only ensured a reduction in medication errors but also improved the patient’s compliance with therapy, self-management, and rebuilt the patient’s confidence in overcoming the disease [47].

The current study reflected a low level of Lebanese CPs’ awareness in adopting the international guidelines. This could be explained by the fact that the Lebanese CPs did not receive any official continuing professional development (CPD) training related to COVID-19. Defined by the Accreditation Council for Pharmacy Education (ACPE), CPE is a “structured educational activity designed or intended to support the continuing development of pharmacists and/or pharmacy technicians to maintain and enhance their competence” [48]. On the other hand, in several countries, community pharmacies have received guidance and support to prepare their staff for the outbreak [4, 49, 50]. Such responses can be used to create regional guidance for pharmacists based on local demands and resources. For example, the Chinese pharmacists’ experience was quite effective in directing and organizing pharmacists’ roles during the COVID-19 pandemic after the prompt release of national and international guidelines [51, 52]. However, in Lebanon, this may not be easy, since the provision of patient care in community pharmacies has historically been plagued by many shortcomings [53]. First, there is a lack of attention at the government level, where the role of the private health sector has often been neglected by national health authorities [32]. This is also applicable to many low to middle-income countries where community pharmacies have been considered as commercial entities instead of healthcare settings, and pharmacists have been perceived as ordinary health workers rather than frontline healthcare professionals [53, 54]. Second, pharmacists have stepped up further responsibilities in the face of the COVID-19 outbreak with a lack of financial incentives [12, 54]. The Lebanese currency lost more than 80% of its value, which in turn affected pharmacists’ income, influencing pharmacists’ motivation and job satisfaction for providing quality pharmaceutical services [18, 55]. Third, pharmacists were placed in a position of combating the pandemic without proper training, thus resulting in inefficient work and low-quality services [3, 53].

Another issue of concern was the pharmacists’ communication with a suspected COVID-19 patient. Our results have shown that non-optimal, ineffective communication was of appreciable concern. These findings were inconsistent with previous SP studies that revealed that the pharmacists’ general communication skills were below expectations [30, 36–38]. Effective communication is a vital component in pharmacists’ daily practice to optimize patient-centered health outcomes. CPs who practice effective communication are more likely to ultimately assess patients’ medical conditions, address their needs, hence improving patients’ confidence and trust in the pharmacy profession [44]. This study highlights the urge for stronger and deeper pharmacist-patient communication with more practical guidance on how to deal with pandemic-related issues.

### Limitations of the study

The recruitment of only one SP throughout the data collection may increase the likelihood of consistency in the delivery of the scenario, the recorded observations, and the grading of the pharmacist’s communication. However, utilizing only one person for documenting several observations at a time, might subject the study to recall bias. Nevertheless, the SP was instructed to complete the observation checklist immediately after each visit to reduce the underreporting of events. It is noteworthy that audio recording the visits would be more efficient in documentation, but this was not feasible in the current study as it is impractical to obtain the participants’ consent before the study conduction because it would influence their actual performance. Furthermore, this study was limited by its relatively small scale, since it was conducted only in Beirut. However, it was not easy to include pharmacies in different Lebanese governorates due to the lockdown and the fear of the SP getting infected by contacting others in the pharmacies. Thus, we recommend a further follow-up study where each pharmacy may be visited twice with two different scenarios, and the SP to be accompanied by another researcher to aid in recording the pharmacists’ responses and reduce the recall bias. This may set a fairer judging criteria for the pharmaceutical services provided.

## Conclusions

Community pharmacists around the world are considered frontline fighters against the COVID-19 pandemic. They are involved in several activities to free up specialized healthcare providers to deal with more serious conditions. This study, however, has revealed an evident unsatisfactory and suboptimal provision of pharmaceutical care to COVID-19 suspects. Moreover, the Lebanese CPs’ communication skills with patients were below expectations. Intensive training for facing the COVID-19 pandemic must be included in the pharmacy curriculum and must be offered to currently practicing pharmacists in Lebanon. Researchers should be engaged in studies with a stronger methodological rigor to thoroughly investigate pharmacists’ intervention, guidance, and support during the pandemic.

## Supplementary Information

**Additional file 1.**

## Data Availability

The dataset presented in this article is available only upon reasonable request since it contains confidential information. Requests to access the datasets should be directed to the corresponding author (r.itani@bau.edu.lb).
