# Supplementary material for: Assessing the pharmaceutical care provision to suspected COVID-19 patients in community pharmacies: a simulated patient study
Source: BMC Health Serv Res. 2022 Apr 9;22:467. doi: 10.1186/s12913-022-07870-1 (PMC8994087; doi:10.1186/s12913-022-07870-1)
Supplement: Supplementary file 1 — Additional file 1. [file 12913_2022_7870_MOESM1_ESM.pdf]

Additional File 1. Data Collection Form

Assessing the Pharmaceutical Care Provision to Suspected COVID-19 Patients in Community Pharmacies: A Simulated Patient Study

Authors: Samar Karout[1], Hani M J Khojah[2], Rania Itani[1*], Fatima Jaffal[1], Abdalla El-Lakany[3]

Form Code: ______

Visiting Information:

Day	Mon.	Year
		**2021**

Hour	Min.	Time
		○ **AM**<br>○ **PM**

Duration Spent (Minutes)

Pharmacy Information

**Code**		**Street**	

Encountered pharmacist Information

Encountered pharmacist gender: ○ Male ○ Female

Pharmacist's estimated age:
○ 22 – 29 years
○ 30 – 39 years
○ 40 – 49 years
○ 50 – 59 years
○ ≥ 60

Method of communication with the pharmacist:
○ Direct contact inside the Pharmacy
○ Through a protection screen (e.g., plexi-glass) inside the pharmacy
○ Through Dispensing window outside the pharmacy

Estimated Average size of the pharmacy:
○ Small (32m²-70m²) ○ Medium (70m²-100m²) ○ Large (>100m²) ○ Could not estimate

Number of staff at the time of visit: _________

Number of customers at the time of visit (inside or outside): ________

Estimated dispensary load:
○ Busy (< 5 customers waiting)
○ Moderate (2–5 customers waiting)
○ Slow (1–2 customers waiting)
○ Quiet (Nil customers)

Relevant medical data asked by the encountered pharmacist

A. Patient Information
☐ Age
☐ Pregnancy/lactation
☐ Occupation (to rule out high-risk occupations, such as HCP, LTCF)
☐ Residence and members sharing living
☐ Medical comorbidities (Do you suffer from any medical disease?)
☐ Medication history (Do you take any chronic medication?)
○ **None of the above**

B. Assessment of COVID-19 Exposure
☐ Close contact with someone with symptoms, diagnosed OR tested positive for COVID-19, in two weeks before feeling sick
☐ Travel history
☐Attending a social gathering, or was in crowded indoor settings (with more than 10 people without universal mask wearing and/or physical distancing)
○ **None of the above**

C. COVID-19 related Symptoms & Management
☐ Nature of symptoms (Can include fever, cough, shortness of breath, diarrhea/GI, headache, muscle ache, chills, sore throat, vomiting, abdominal pain, nasal congestion, loss of smell, loss of taste, malaise, and fatigue)
☐ Onset and duration of symptoms (When did the symptoms start?)
☐ Life-threatening symptoms (red flag symptoms, such as severe dyspnea, persistent chest pain, new confusion, inability to wake up or stay awake, bluish lips or face)
☐ Medications used to cope with those symptoms (Have you tried any treatment?)
☐ Previous PCR test (date and results of PCR, if applicable)
☐ History of Flu vaccination
☐ Others, ________________________
○ **None of the above**

Pharmacist Responses to the patient's situation (Select all that apply):

First Response:
○ Advised to do a PCR test

Medical Attention:
○ Advised to seek medical attention
○ Referred to a specific healthcare facility, e.g. private clinic having a contract with the pharmacy

Other Responses:
☐ Asked the patient to leave the pharmacy and refused to talk
☐ Called the Ministry of Health (MOPH)
☐ Stated that he/she cannot help
☐ Advised the patient to isolate herself for 14 days
☐ Measured the patient's body temperature
☐ Emphasized on the importance of avoiding the use of antimicrobials (e.g. antibiotics, antivirals) without a prescription
☐ Requested a prescription to sell any claimed medications for COVID-19
☐ Educated about COVID-19, typical symptoms and measures to prevent transmission
☐ Advised to stick to PPE (mask, disinfectant, gloves…)
☐ Recommended non-pharmacological measure(s): ______________________________________
☐ Advised to monitor red-flag symptoms
☐ Recommended an antipyretic for fever
☐ Recommended other medication(s)
☐ Recommended a dietary supplement
☐ Recommended a flu vaccine
☐ Asked the patient to keep a distance from the dispensary counter
☐ Asked the patient to take distance from other clients present in the pharmacy
☐ Started disinfecting the area once he/she suspected about a COVID-19 case
☐ Informed patients and other staff about the suspected case
☐ Other related responses: ____
○ **None of the above**

If recommended a medication: (Select all that apply)

Medications prescribed:

☐ Antibiotic
☐ Antiviral
☐ Antihistamine
☐ Pseudoephedrine
☐ Corticosteroids
☐ Antitussive

Antipyretic

○ Paracetamol
○ NSAIDs

Dietary Supplements

☐ Vitamin D
☐ Vitamin C
☐ Zinc
☐ Vitamin E
☐ Vitamin B complex

Intranasal products

☐ Intranasal Saline
☐ Intranasal Corticosteroids
☐ Intranasal Decongestant
☐ Intranasal Antihistamine

Others: ______________________________

First medication (Specify the medication being recommended):

Brand Name:
Dose:
Dosage form:

*** Information being provided by the pharmacist about the recommended medication**

☐ Indication; ____________________________
☐ Mode of action; ________________________
☐ Dosage regimen: _______________________
☐ Duration of use; ________________________
☐ Side effects: ___________________________

Second medication (Specify the medication being recommended):

Brand Name:
Dose:
Dosage form:

*** Information being provided by the pharmacist about the recommended medication**

☐ Indication; ____________________________
☐ Mode of action; ________________________
☐ Dosage regimen: _______________________
☐ Duration of use; ________________________
☐ Side effects: ___________________________

Pharmacist's Communication Skills

Category	1	2	3	4	Score
**Eye contact with patient**	Made no eye contact	Made some eye contact	Made eye contact, but disengaged several times	Maintained appropriate eye contact throughout interview	
**Nonverbal communication**	Leaned away from patient with arms crossed	Leaned away from patient	Leaned toward patient, but was either too far or too close to patient	Leaned toward patient from a safe distance	
**Listening**	Appeared to be consistently distracted	Was occasionally distracted	Was not distracted, but did not seem to be fully engaged with patient	Listened actively to patient at all times	
**Questions**	Asked appropriate questions	Asked appropriate questions, but none were open-ended	Asked appropriate, open-ended questions, but some were not understandable	Asked questions which were appropriate, open-ended, and understandable	
**Concern**	Appeared hurried and/or not interested in patient	Took the necessary time, but did not seem interested in the patient	Showed some interest in the patient but inconsistently	Showed consistent interest and concern towards the patient	
**Organization**	Seemed totally unprepared	Seemed prepared, but carried out interview in a random manner	Seemed prepared and somewhat sequential	Demonstrated a prepared, well-organized, sequential approach to the interview	
**Empathy**	Showed no interest in the patient's emotional needs	Showed interest in the patient's emotional needs, but did not respond to them	Responded to the patient's emotional needs, but lacked warmth and sincerity	Demonstrated appropriate, sincere interest in the patient's emotional needs	
**Closure**	Ended interview abruptly	Effectively ended interview, but did not summarize patient's concerns	Summarized patient's concerns, but did not ask if patient had any other concerns or questions	Summarized patient's concerns and asked if patient had any other concerns or questions	
**Total Score**					
